# Role of Mean Platelet Volume in Diagnosis of Childhood Acute Appendicitis

**DOI:** 10.1155/2012/823095

**Published:** 2012-08-27

**Authors:** Bunyamin Uyanik, Cemil Kavalci, Engin Deniz Arslan, Fevzi Yilmaz, Ozgur Aslan, Serdal Dede, Fatih Bakir

**Affiliations:** ^1^Emergency Department, Numune Training and Research Hospital, 06130 Ankara, Turkey; ^2^Biochemistry Department, Numune Training and Research Hospital, 06130 Ankara, Turkey

## Abstract

*Introduction*. Acute appendicitis is the leading cause of abdominal pain in children requiring emergency surgical intervention. The aim of this study is to investigate the diagnostic value of MPV in early diagnosis of acute appendicitis cases in pediatric age group. *Methods*. This study was performed retrospectively. Three hundred five patients operated on with the diagnosis of appendicitis and pathologically found to be acute appendicitis were classified as Group 1 and 305 healthy children were classified as control Group 2. *Results*. One hundred ninety-seven of 305 cases in Group 1 are males (64.6%), in Group 2, 151 of 305 cases are males (49.5%). The mean MPV in Group 1 was 7.9 ± 0.9 (fL), and whereas in Group 2 was 7.7 ± 0.8 (fL). There was no statistically significant difference regarding MPV values (*P* > 0.05). *Conclusion*. In our study we detected that mean platelet volume has no diagnostic value in pediatric acute appendicitis cases.

## 1. Introduction 

Acute appendicitis is the leading cause of abdominal pain in children requiring emergency surgical intervention. In pediatric patients suspected with acute appendicitis, the negative laparotomy ratio is higher than that of adults. This is mainly because nonsurgical diseases of childhood such as gastroenteritis, constipation, infantile colic, drug poisoning, uremia, diabetic ketoacidosis, mesenteric lymphadenopathy, lower respiratory tract infections, and urinary tract infections are mimicking acute abdomen. Limitations in obtaining anamnesis from children, maladaptiveness to physical examination, and child's inability to converse increase diagnostic difficulty even more. Delays in diagnosis lead to complications such as perforation, abscess, peritoneal inflammation, sepsis, intestinal obstruction [1–4]. 

Mean platelet volume (MPV) is a parameter calculated and provided by automatic blood count equipment during routine blood counts. Although MPV is not generally taken into consideration by clinicians; it could be a marker of platelet activation, because large platelets are more reactive, produce more prothrombotic factors, and aggregate more easily [5]. A subject of investigation since 1970s, MPV's relationship with thrombocyte function and activation is known [6–9]. In many researches conducted recently MPV evaluated in the diagnosis and followup of many diseases such as infectious endocarditis, helicobacter pylori gastritis, Behcet disease, ulcerative colitis (UC), rheumatoid arthritis, psoriatic arthritis, Henoch-Schönlein purpura (HSP) progressing with inflammation, and ischemic diseases like acute ischemic stroke, acute myocardial infarction, and pulmonary thromboembolism [10–19].

The aim of this study is investigation of diagnostic value of MPV in early diagnosis of acute appendicitis cases in pediatric age group. 

## 2. Methods

This study was conducted retrospectively after obtaining an approval from the Numune Training and Research Hospital ethical committee. Polyclinic and admission files as well as automation system records of patients admitted to hospital between January 2006 and January 2010 were investigated. Exclusion criteria is shown on the Table 1. MPV was determined on admission, collected into ethylenediaminetetraacetic acid (EDTA) tubes, and processed within 1 hour after venipuncture.

In this period, the files and records of 536 cases surgically operated on with the diagnosis of acute appendicitis were reached. One hundred eighty-one of these cases were excluded from this study due to missing information or data. Pathology results of 305 cases out of 355 included in the study were reported as acute appendicitis. 

Three hundred five patients operated on with the diagnosis of appendicitis and pathologically found to be acute appendicitis were classified as Group 1 and 305 healthy children without any active complaint, chronic disease, and with normal physical examination results who were brought for checkups to the hospital's child polyclinic were classified as control Group 2.

Age, gender, leukocyte and thrombocyte counts at the time of hospital admission, MPV values, pathology results of patients operated on with acute appendicitis diagnosis, and healthy children's age, gender, leukocyte and thrombocyte counts at the time of hospital admission, and MPV values were recorded. 

Data of both groups were statistically compared. SPSS for Windows 13.0 software was used in statistical analysis. The patients demographic and clinical features are shown as mean ± standard deviation, median, range, and percentage (%). The normal distributions were tested with Kolmogorov-Smirnov test. Student's  *t*-tests were used in two continuous group comparisons. An alpha value of less than *P* < 0.05 was considered statistically significant.

## 3. Results

In the study, 610 subjects were evaluated in two groups. Group 1 consisted with acute appendicitis whereas Group 2 was control group. 

 One hundred ninety-seven of 305 cases in Group 1 are males (64.6%), and in Group 2, 151 of 305 cases are males (49.5%). The sociodemographic characteristics of the patients and the control group are summarized in Table 2. There was no statistically significant difference among the groups with respect to gender and age (*P* > 0.05). 

 The mean leukocyte count in Group 1 was 11610 ± 5430 (/*μ*L), whereas in Group 2 was 8138 ± 2285 (/*μ*L). There was a statistically significant difference among the groups in terms of leukocyte (*P* < 0.05). 

The mean MPV value in Group 1 was 7.9 ± 0.9 (fL), whereas in Group 2 was 7.7 ± 0.8 (fL). There was no statistically significant difference according to MPV values (*Z* = − 0.63, *P* > 0.05).

## 4. Discussion

Although acute appendicitis is the best known entity in terms of classical symptomatology and examination findings, it still protects its ranking among the causes of abdominal pain most difficult to diagnose. 

Platelet activation is a link in the pathophysiology of diseases prone to thrombosis and inflammation. Numerous platelet markers, including mean platelet volume (MPV), have been investigated in association with both thrombosis and inflammation. High MPV associates with different established risk factors, cardio- and cerebrovascular disorders, and low-grade inflammatory conditions prone to arterial and venous thromboses. High-grade inflammatory diseases, such as active rheumatoid arthritis or attacks of familial Mediterranean fever, present with low levels of MPV, which reverse in the course of anti-inflammatory therapy [20]. Instead of MPV decreasing in acute events, MPV increases in chronic events.

In recent studies, a relationship between the rise of leukocyte count and appendicitis diagnosis have been shown. It has been reported that in patients with acute appendicitis, the sensitivity of leukocyte is between 60–87%, and specificity 53–100% [13, 21]. Its increase with CRP can assist diagnosis with a sensitivity reaching 98%. It has been shown that leukocyte count increases during the early stages of the disease and prior to perforation, but no statistically significant difference exists between leukocyte counts of patients with perforated appendicitis and those without perforation [21–26]. Our findings concur with the literature. We found that the leukocyte has increased in response to the infection in acute appendicitis group. 

Increased platelet volume has been widely accepted as an independent risk factor in coronary artery diseases and cerebrovascular diseases [27–29]. Slavka et al. reported subjects with higher MPV (>11.01 fL) value had 1.5 times higher vascular mortality risk than in patients with low MPV (<8.7 fL) value. In the same study, significant positive relationship between high MPV and the risk of ischemic heart disease has been identified [30]. The increase in the mean platelet volume is considered to be a good indicator of higher thrombocyte activity.

Albayrak et al. showed low MPV to be statistically significant in their study which compares adult acute appendicitis cases with healthy adult control group [31]. They have reported that MPV should not be ignored during the diagnostic stages of patients with suspected acute appendicitis. Bilici et al. have reported significant reduction in MPV in pediatric age group acute appendicitis events in comparison to the control group [32]. But in our study no statistically significant relationship between MPV and pediatric appendicitis was established. Frequency of inflammatory events in pediatric ages led by infections and its difficulty of identification may have caused overlooking of the presence of inflammatory diseased cases in healthy child group, and this may have caused lower MPV and erroneous statistics. Intense immune complexes in acute inflammation can cause destruction of erythrocytes and may cause fragmented cells to be counted as thrombocytes. Thrombocyte counts which is not confirmed by peripheral smear may be interpreted as false MPV decrease. The smaller number of sample size of previous studies may be the cause of difference of MPV results. When the sample size increases, cross-sectional errors will decrease.

## 5. Conclusion

In our study we detected that mean platelet volume has no diagnostic value in pediatric acute appendicitis cases. In order to clarify the subject, a wide event series and prospective research is needed.

## Figures and Tables

**Table 1:** Exclusion criteria.

Age >18 year
Diabetes mellitus, hypertension, obesity, and comorbidity

**Table 2:** Demographics and laboratory findings of patients.

	Group 1	Group 2	*P*
Sex (%)			
Male	65	49	>0.05
Female	35	51	
Age (year) (mean ± SD)	9.5 ± 2.9	9.6 ± 3.1	>0.05
Leukocyte (/*μ*L) (mean ± SD)	16100 ± 5430	8138 ± 2285	<0.05
MPV (fL) (median, IQR)	7.60 (1.10)	7.55 (1.00)	>0.05

MPV: mean platelet volume.
